# A coarsened multinomial regression model for perinatal mother to child transmission of HIV

**DOI:** 10.1186/1471-2288-8-46

**Published:** 2008-07-15

**Authors:** Charlotte C Gard, Elizabeth R Brown

**Affiliations:** 1 Department of Biostatistics, University of Washington, Seattle, WA, USA

## Abstract

**Background:**

In trials designed to estimate rates of perinatal mother to child transmission of HIV, HIV assays are scheduled at multiple points in time. Still, infection status for some infants at some time points may be unknown, particularly when interim analyses are conducted.

**Methods:**

Logistic regression models are commonly used to estimate covariate-adjusted transmission rates, but their methods for handling missing data may be inadequate. Here we propose using coarsened multinomial regression models to estimate cumulative and conditional rates of HIV transmission. Through simulation, we compare the proposed models to standard logistic models in terms of bias, mean squared error, coverage probability, and power. We consider a range of treatment effect and visit process scenarios, while including imperfect sensitivity of the assay and contamination of the endpoint due to early breastfeeding transmission. We illustrate the approach through analysis of data from a clinical trial designed to prevent perinatal transmission.

**Results:**

The proposed cumulative and conditional models performed well when compared to their logistic counterparts. Performance of the proposed cumulative model was particularly strong under scenarios where treatment was assumed to increase the risk of in utero transmission but decrease the risk of intrapartum and overall perinatal transmission and under scenarios designed to represent interim analyses. Power to estimate intrapartum and perinatal transmission was consistently higher for the proposed models.

**Conclusion:**

Coarsened multinomial regression models are preferred to standard logistic models for estimation of perinatal mother to child transmission of HIV, particularly when assays are missing or occur off-schedule for some infants.

## Background

In trials designed to evaluate the efficacy of an intervention to prevent perinatal mother to child transmission (PMTCT) of human immunodeficiency virus (HIV), infants are usually tested within 48 hours after birth, with a second visit scheduled 4 to 8 weeks after birth, to determine their HIV status. Test results from these two visit windows are used to ascertain the three main outcomes of scientific interest:

A1 The probability of in utero transmission, estimated by the fraction of infants testing positive at birth;

A2 The probability of perinatal transmission, estimated by the fraction of infants testing positive by 8 weeks;

A3 The probability of intrapartum transmission, estimated by the fraction of infants testing positive by 8 weeks who tested negative at birth.

Subsequent visits may take place (i.e., after 8 weeks) but tests at these visits only contribute information about the outcomes of interest if the infant has missed earlier scheduled visits.

In primary analyses, we are usually interested in obtaining unadjusted estimates of A1, A2, and A3. In secondary analyses, covariate-adjusted estimates are often desired. If every infant was tested in every visit window, we could use a binary endpoint approach such as logistic regression to obtain adjusted estimates for each of the three main outcomes. However, missed and off-schedule visits are not uncommon in PMTCT trials. And, even if there are no missed visits, interim analyses may occur when only a fraction of the infants are old enough for the second visit.

Table 1 lists a selection of primary papers from trials aimed at reducing PMTCT of HIV and summarizes their methods for unadjusted and adjusted analysis. These methods are among the more commonly used for estimating PMTCT of HIV. In adjusted analyses, the endpoint is generally modeled as either binary or right-censored continuous using logistic or Cox proportional hazards (PH) regression, respectively. For both logistic and Cox PH models, methods currently used for handling missing data may be inadequate. For example, when the logistic model is used and a test result is missing for an infant who has not previously tested positive, the observation is dropped, although if subsequent tests are negative, the missing test result may be imputed to be negative. When the Cox PH model is used, an infant's time to HIV infection is right censored at his or her last negative test; however, approaches for addressing timing of infection when a missing visit is followed by a positive test and there have been no previous positive tests may be inadequate. Some authors use the time of the first positive test as the time of infection while others use the midpoint between the last negative and first positive tests (or birth and the first positive test). Cox PH models do not specifically address treatment effects on A1–A3 but instead estimate the average treatment effect over the observation period. While we generally think of using Cox PH models to model time to an event, in the case of PMTCT, the event of interest has already occurred (or not occurred) when an infant is first tested. Because of the imperfect sensitivity of the test, however, we may not have been able to detect it.

**Table 1:** Approaches taken in selected papers analyzing PMTCT of HIV.

	Unadjusted	Adjusted	Censoring
Wiktor et al. [4]	KM^1^	-	at last negative test
Guay et al. [16]	KM	PH^2^	at last negative test
Dabis et al. [17]	KM	PH	not stated
Shaffer et al. [6]	KM	logistic regression	not stated
Kuhn et al. [18]	KM	PH and logistic regression	at last follow-up
Fawzi et al. [19]	Chi-square tests	PH	not stated
Dorenbaum et al. [7]	Fisher's exact tests	logistic regression	-
Moodley et al. [8]	KM	PH and logistic regression	at last follow-up
Magder et al. [2]	likelihood approach	logistic regression (coarsened data)	-

^1^Kaplan-Meier. ^2^Cox proportional hazards.

We propose a coarsened multinomial regression model for analyzing PMTCT of HIV that accommodates missing and off-schedule test data and allows the effect of treatment to depend upon time. The approach is motivated by the HIV Prevention Trials Network (HPTN) 024 study, a multi-site placebo-controlled trial of antiobiotics to prevent chorioamnionitis and, therefore, PMTCT of HIV. In HPTN 024, of the 2,052 liveborn infants, 1,813 (88%) had HIV tests within 48 hours of delivery and 1,696 (83%) had tests 4 to 8 weeks after delivery. Only 1,584 (77%) had tests both within 48 hours of delivery and 4 to 8 weeks after delivery. While missed visits were sometimes due to the infant's death, in some cases the mother simply forgot or was unable to bring the infant in for follow-up. Many mothers did not deliver at the study hospital and had to bring their infants in later for the birth HIV test, resulting in visits that occurred off schedule.

Bertolli et al. [1] use a coarsened data approach to estimate unadjusted rates of in utero and intrapartum transmission based on the assumption that infants with missing test data are distributed among transmission groups in the same proportions as infants with non-missing test data. Magder et al. [2] expand on this approach, using logistic regression to estimate covariate-adjusted associations between various risk factors and presumed time of transmission, while allowing for misclassification. Little and Rubin [[3], pp. 169–70] describe an approach for estimating the parameters of a coarsened multinomial model using the Expectation Maximization algorithm. While it addresses the problem of incomplete data, it does so only for a single sample or independent samples. Here we allow for regression models of the probabilities for the three outcomes of interest. We begin by describing the coarsened multinomial model then lay out strategies to adjust for covariates. Next, we describe a simulation study designed to evaluate the performance of the proposed regression estimators and compare them to more commonly used approaches in PMTCT trials. We illustrate our approach with an analysis of HPTN 024 study data then follow with discussion and conclusions.

## Methods

### The coarsened multinomial model

In this section, we present the coarsened multinomial model. In this general presentation, we assume that there are *J *visit windows. Usually, when estimating PMTCT, *J *= 2, corresponding to birth and 4 to 8 weeks. However, depending upon the study design, *J *may be larger as in Wiktor et al. [4], where the main endpoint was infection status at three months, and infants were tested at birth, four weeks, and three months.

We begin by dividing the follow-up time into windows as follows:

$$\begin{array}{cc} \begin{array}{r} \text{First visit window}\\ \text{Second visit window}\\ \vdots \\ \text{Time following last visit window} \end{array} & \begin{array}{l} \left[t_{11},t_{12}\right)\\ \left[t_{21},t_{22}\right)\\ {}[t_{J+1,1},\infty ). \end{array} \end{array}$$

Here *t*_*j*1 _and *t*_*j*2 _indicate the times at which the *j*th visit window starts and ends, respectively. These intervals do not have to be and usually are not contiguous. In other words, *t*_*j*2 _is not necessarily equal to *t*_*j*+1,1_. Unscheduled or off-schedule visits result in tests that occur in the interval [*t*_*j*2_, *t*_*j*+1,1_).

We define a complete response vector for the *i*th, *i *= 1,..., *N*, infant as $Y_{i}^{*}=(Y_{i1}^{*},...,Y_{i,J+1}^{*}{)}^{\prime }$, where $Y_{ij}^{*}$ = 1, *j *= 1,..., *J *+ 1, if the infant tests positive for the first time at the *j*th visit and 0 otherwise. The vector $Y_{i}^{*}$ represents a multinomial response as the *i*th infant can only test positive for HIV for the first time once. When an infant misses a scheduled visit (including if he or she is late or early for the visit), we observe a coarsened version of $Y_{i}^{*}$, which we denote *Y*_*i *_= (*Y*_*i*1_,..., *Y*_*i*,*J*+1_)*'*. Effectively, *Y*_*ij *_= 1 for all the windows in which the infant may have first become positive. More formally, taking $t_{i}^{p}$ and $t_{i}^{n}$ to be the time of the first positive test result for infant *i *and the time of the last negative test result for infant *i*, respectively, we define *Y*_*i *_as

(1) $$Y_{i1}=\{\begin{array}{ll} 1, & t_{i}^{p}\in [0,t_{12})\\ 1, & t_{i}^{p}\geq t_{12}\text{ and }t_{i}^{n}<t_{11}\\ 0, & \text{otherwise} \end{array}$$

(2) $$Y_{ij}=\{\begin{array}{cc} \begin{array}{ll} 1, & t_{i}^{p}\in [t_{j-1,2},t_{j2})\\ 1, & t_{i}^{p}\geq t_{j2}\text{ and }t_{i}^{n}<t_{j1},\\ 0, & \text{otherwise} \end{array} & j=2,...,J, \end{array}$$

(3) $$Y_{i,J+1}=\{\begin{array}{ll} 1, & t_{i}^{p}\geq t_{J2}\\ 0, & \text{otherwise}. \end{array}$$

For infants with no positive test result during follow-up, we take $t_{i}^{p}$ = ∞. For infants with no negative test result during follow-up, we take $t_{i}^{n}$ = -∞. We assume that each infant has at least one (non-missing) test result during the follow-up period.

To illustrate how the observed vector *Y *relates to the unobserved but true outcome Y*, we look at two possible visit and outcome patterns. First, we examine the effect of a missed visit assuming *J *= 2 visit windows. We consider infant **A**, who was not tested until the second visit at which point he or she tested positive. We do not know if infant **A **would have tested positive had he or she come in for the first visit. We can say, however, that the infant would have tested positive for the first time at the first visit or at the second visit if tested at both. In other words, Y* for this infant may be (1, 0, 0)*' *or (0, 1, 0)*' *but is not (0, 0, 1)*'*. Therefore, by equations (1), (2), and (3), *Y *= (1, 1, 0)*'*. Next, we consider infant **B **who missed the first visit and tested negative at the second visit. We assume that if an infant tests negative at the end of the study he or she was negative throughout the study; therefore, Y* = (0, 0, 1)*' *and *Y *= (0, 0, 1)*' *for this infant. In this case, even though the infant was not tested in every visit window, we have complete information regarding his or her outcome. This illustrates one difficulty involving missed visits. If a infant is uninfected and misses all visits except the last (the *J*th visit), we still have complete information about him or her (as in **B**); however, if the infant is infected at the last visit (as in **A**), we have incomplete information about him or her.

#### Modeling cumulative rates of transmission

We begin by considering regressions on cumulative probabilities in order to estimate endpoints A1 and A2, the in utero and perinatal transmission rates. We define the probability that the *i*th infant's first positive test occurs in the *j*th visit window as *π*_*ij *_and the probability that the *i*th infant's first positive test occurs after the last visit window as $\pi_{i,J+1}=1-\sum_{j=1}^{J}\pi_{ij}$. To examine the relationship between a set of predictors, (*X*_*i*1_,..., *X*_*im*_), and the probability that infant *i *tests positive at or before the *j*th visit, we define the following regression model:

(4) $$g(\pi_{i1})={X^{\prime }}_{i}\beta_{1}$$

(5) $$\begin{array}{cc} g(\sum_{k=1}^{j}\pi_{ik})={X^{\prime }}_{i}\beta_{j}, & j=2,...,J. \end{array}$$

where *g*(·) is a link function that specifies the relationship between the predictors *X*_*i *_= (1, *X*_*i*1_,..., *X*_*im*_)*' *and the response, through the parameter vector *β*_*j *_= (*β*_*j*0_,..., *β*_*jm*_)*' *of length m+1. For ease of exposition, we assume that a predictor is relevant for all visit windows; however, this is not necessary as will be illustrated in the HTPN 024 analysis.

When modeling cumulative rates or probabilities, two appropriate choices for the link function are the log link, where *g*(*p*) = log(*p*), and the logit link, where *g*(*p*) = logit(*p*) = log{*p*/(1 - *p*)}. When using the logit (log) link, *β*_*jl*_, *l *= 1...,*m*, is interpreted as the log odds ratio (log relative risk) for testing positive at or before the *j*th visit window per one unit increase in *X*_*il*_, *l *= 1,..., *m*.

We combine and re-write equations (4) and (5) to obtain the following expressions for *π*_*ij*_:

$$\pi_{i1}=g^{-1}({X^{\prime }}_{i}\beta_{1})$$

$$\begin{array}{cc} \pi_{ij}=g^{-1}({X^{\prime }}_{i}\beta_{j})-g^{-1}({X^{\prime }}_{i}\beta_{j-1}), & j=2,...,J. \end{array}$$

The log-likelihood, written in terms of the coarsened data, is given by

(6) $$l(\pi_{i1},...,\pi_{iJ})=\sum_{i=1}^{N}\log\left[\sum_{j=1}^{J}Y_{ij}\pi_{ij}+Y_{i,j+1}(1-\sum_{j=1}^{J}\pi_{ij})\right],$$

and maximum likelihood methods are used to estimate the parameters (see Maximum likelihood estimation of parameters below).

#### Modeling conditional rates of transmission

We now consider regressions on conditional probabilities to estimate endpoint A3, the intrapartum transmission rate. To examine the relationship between a set of predictors (*X*_*i*1_,..., *X*_*im*_) and $\pi_{ij|{\left(j-1\right)}^{-}}=\frac{\pi_{ij}}{1-\sum_{k=1}^{j-1}\pi_{ik}}$, the conditional transmission rate or probability of first testing positive at the *j*th visit given a negative test result at the (*j *- 1)th visit, we define the following regression model:

$$g(\pi_{i1})={X^{\prime }}_{i}\beta_{1}$$

$$\begin{array}{cc} g(\pi_{ij|{\left(j-1\right)}^{-}})={X^{\prime }}_{i}\beta_{j|{\left(j-1\right)}^{-}}, & j=2,...,J, \end{array}$$

where *g*(·) is a link function that specifies the relationship between the predictors *X*_*i *_= (1, *X*_*i*1_,..., *X*_*im*_)*' *and the response, through the parameters vectors *β*_1 _= (*β*_10_,..., *β*_1*m*_)*' *and $\beta_{j|{\left(j-1\right)}^{-}}=(\beta_{j|{\left(j-1\right)}^{-},0},...,\beta_{j|{\left(j-1\right)}^{-},m}{)}^{\prime }$, each of length *m *+ 1. When using the logit (log) link, $\beta_{j|{\left(j-1\right)}^{-},l}$, *l *= 1..., *m*, is interpreted as the log odds ratio (log relative risk) for testing positive at the *j*th visit, given a negative result at the (*j *- 1)th visit, per one unit increase in *X*_*il*_, *l *= 1,..., *m*.

We calculate *π*_*ij*_, *j *= 2,..., *J*, for use in the log-likelihood as

$$\pi_{ij}=\pi_{ij|{\left(j-1\right)}^{-}}\times (1-\sum_{k=1}^{j-1}\pi_{ik}),$$

where

$$\pi_{ij|{\left(j-1\right)}^{-}}=g^{-1}({X^{\prime }}_{i}\beta_{j|{\left(j-1\right)}^{-}}).$$

Equation (6) then provides the likelihood for the coarsened data.

#### Maximum likelihood estimation of parameters

To obtain maximum likelihood estimates of the regression parameters, we maximize equation (6) using numerical optimization techniques. The optimization procedure requires that *π*_*i*1_,..., *π*_*iJ *_lie between 0 and 1 and that $\sum_{j=1}^{J}\pi_{ij}$ be less than 1 for all *i*. If these constraints are not met by the form of the regression models, we impose them through the optimization procedure via non-linear constraints on the coefficients. Further implications of the constraints are presented in the discussion.

For the analyses presented here, numerical optimization was carried out using a quasi-Newton algorithm. The algorithm is an efficient modification of Powell's Variable Metric Constrained WatchDog algorithm, which is available in SAS PROC NLP [5]. Additional details regarding our implementation, including SAS macros for fitting the cumulative and conditional models with the logit link for an arbitrary number of visit windows, are available from the authors upon request.

### Simulation study

We performed simulations to assess the properties of the proposed regression estimators and compare them to more commonly used regression approaches. We considered the case of two visit windows corresponding to birth and 4 to 8 weeks. For each simulated data set, we randomly generated covariates that informed the infant's simulated mode of transmission (in utero, during delivery, or neither). We allowed for imperfect sensitivity of the assay shortly after transmission by simulating time of detectable infection and allowed the simulations to reflect additional positive test results at the 4 to 8 week visit due to breastfeeding. We randomly generated a set of visit times for each infant, independent of covariates but dependent upon the endpoint, with infants having simulated time of detectable infection equal to 0 days slightly more likely to attend the 4 to 8 week visit. We determined each infant's observed endpoints by comparing his or her simulated time of detectable infection to his or her simulated visit times. Additional details regarding the simulation of time of detectable infection and visit process are provided in Appendices A and B (Additional files 1 and 2), respectively.

We fit the cumulative and conditional regression models using standard logistic regression and the proposed coarsened multinomial (CM) regression models with the logit link. We considered two sets of logistic models: the first (L-CUM) modeled infection at birth and infection at 4 to 8 weeks, and the second (L-COND) modeled infection at birth and infection at 4 to 8 weeks among infants known to be HIV negative at birth. The logistic models considered all infants for whom HIV status at birth and HIV status at 4 to 8 weeks could be determined and were chosen to represent those commonly used in the analysis of PMTCT of HIV [6-8]. Cox PH models, although also used, do not specifically address treatment effects on A1–A3 but instead estimate the average effect of treatment over the observation period; therefore, we did not assess them in our simulations. We compared the effects of treatment as obtained from the regression models to the true effects of treatment according to which the data were generated, determining bias, mean squared error (MSE), 95 percent coverage probability (CP), and power for each estimator. We considered several scenarios, allowing for different treatment effects (TEs) and different visit processes (VPs) that resulted in varying amounts of missing data. Results are provided for each scenario based on 1000 data sets of 1500 observations each.

In carrying out numerical optimization, we chose the convergence criteria for the proposed cumulative and conditional regression models to coincide with the convergence criteria for the logistic regression models in SAS PROC LOGISTIC [9].

### HPTN 024

To illustrate our approach, we analyzed data from HPTN 024, a multi-site double-blinded placebo controlled trial of antiobiotics to prevent chorioamnionitis and, therefore, perinatal transmission of HIV. The trial enrolled pregnant, HIV positive women receiving care in hospitals and clinics in Malawi, Tanzania, and Zambia. Women were randomized to receive either treatment or placebo. Treatment consisted of two courses of antibiotics, with the first course administered at enrollment (20 to 24 weeks gestation) and the second at the onset of contractions and/or premature rupture of membranes. All women and their liveborn infants were offered single dose nevirapine per World Health Organization recommendation [10]. Women were followed during their pregnancies, and their infants were followed postnatally. Visit windows for determining in utero and delivery/early postnatal transmission in this breastfeeding population were 0 to 48 hours and 4 to 6 weeks, respectively. Because over half of the visits scheduled to occur between 4 and 6 weeks actually took place between 6 and 8 weeks, we extended the second visit window to 4 to 8 weeks for analysis purposes. We also extended the birth visit window to 0 to 7 days.

At the first interim analysis, the NIAID Vaccine and Prevention Data and Safety Monitoring Board reviewed trial progress in a scheduled interim analysis and concluded that, while statistical evidence neither established benefit nor harm, the available evidence ruled out targetted levels of benefit. They further recommended that HPTN 024 stop recruitment and administration of study drug and continue follow-up of enrolled women and infants. Administration of the study drug was halted on March 5, 2003. Additional details regarding the 024 study are provided by Taha et al. [11].

In this analysis, we examined the association between PMTCT and antibiotics, comparing outcomes for infants born to mothers randomized to antibiotics who delivered prior to March 5, 2003 to infants born to mothers randomized to placebo or to mothers randomized to antibiotics who delivered after March 5, 2003. Additional covariates of interest were log maternal viral load, maternal CD4 count, and infant gender. In the birth model, we adjusted for mother's use of nevirapine and, in the 4 to 8 week model, for mother's and infant's use of nevirapine. To account for unmeasured differences between hospitals and clinics, we included study site in both models.

## Results

### Simulation study

Table 2 provides simulation results for the six combinations of treatment effect and visit process that we examined. These combinations illustrate the impact of treatment effect on estimator performance for a given visit process as well as the impact of visit process (i.e., varying levels of missingness) on estimator performance for a given treatment effect. Because we allowed for imperfect sensitivity and early breastfeeding transmission, we would not expect to see zero bias in the estimates from our simulations. Relative bias (not shown in table) of estimators of treatment effect on perinatal and intrapartum transmission ranged from 0.007 (conditional CM model) to 0.711 (cumulative logistic model), corresponding to the scenario where treatment was assumed to increase the risk of in utero transmission but decrease the risk of intrapartum and overall perinatal transmission (TE4) and the visit process resulted in the most missing data (VP3).

**Table 2:** Simulation results for six treatment effect (TE)/visit process (VP) scenarios.

	Bias		MSE		CP		Power	
				
Scenario	A1	A2/A3	A1	A2/A3	A1	A2/A3	A1	A2/A3
TE1/VP1 (*β*_11 _= -0.55, *β*_21 _= *β*_2|1^-^,1_ = -0.54)								

L-CUM	0.013	0.045	0.069	0.030	0.948	0.943	0.518	0.845
CM-CUM	0.027	0.048	0.068	0.027	0.948	0.939	0.499	0.882
L-COND	-0.015	0.107	0.077	0.077	0.959	0.914	0.539	0.439
CM-COND	-0.007	0.106	0.072	0.072	0.956	0.901	0.541	0.461

TE2/VP1 (*β*_11 _= -0.55, *β*_21 _= *β*_2|1^-^,1_ = 0)								

L-CUM	0.173	-0.020	0.096	0.022	0.879	0.952	0.309	
CM-CUM	0.168	-0.009	0.094	0.020	0.873	0.952	0.339	
L-COND	0.098	0.004	0.083	0.050	0.938	0.956	0.398	
CM-COND	0.101	0.006	0.079	0.046	0.941	0.952	0.405	

TE3/VP1 (*β*_11 _= -0.02, *β*_21 _= *β*_2|1^-^,1_ = -0.38)								

L-CUM	-0.078	0.055	0.058	0.029	0.952	0.931	0.057	0.517
CM-CUM	-0.064	0.049	0.054	0.025	0.954	0.933	0.056	0.605
L-COND	-0.050	0.064	0.062	0.059	0.946	0.941	0.062	0.263
CM-COND	-0.042	0.063	0.058	0.055	0.944	0.944	0.057	0.264

TE4/VP1 (*β*_11 _= 0.27, *β*_21 _= *β*_2|1^-^,1_ = 0.27)								

L-CUM	-0.123	0.059	0.065	0.028	0.921	0.936	0.096	0.271
CM-CUM	-0.105	0.049	0.057	0.023	0.931	0.942	0.111	0.307
L-COND	-0.043	0.034	0.058	0.060	0.953	0.937	0.160	0.173
CM-COND	-0.038	0.033	0.055	0.056	0.954	0.936	0.186	0.187

TE4/VP2								

L-CUM	-0.110	0.116	0.064	0.045	0.927	0.898	0.103	0.138
CM-CUM	-0.096	0.043	0.059	0.032	0.927	0.940	0.128	0.261
L-COND	-0.073	0.041	0.061	0.084	0.941	0.949	0.138	0.123
CM-COND	-0.066	0.042	0.054	0.078	0.946	0.945	0.146	0.134

TE4/VP3								

L-CUM	-0.124	0.192	0.113	0.105	0.923	0.890	0.068	0.064
CM-CUM	-0.088	0.017	0.097	0.058	0.939	0.954	0.096	0.193
L-COND	-0.054	-0.009	0.099	0.292	0.944	0.967	0.095	0.077
CM-COND	-0.040	0.002	0.087	0.211	0.946	0.964	0.102	0.086

TE scenarios are defined by *β*_11_, *β*_21_, and *β*_2|1^-^,1_, which represent the true log odds ratios for in utero transmission (A1), perinatal transmission (A2), and intrapartum transmission (A3), respectively, comparing treatment versus control (see Appendix A). VP scenarios are defined such that the amount of missing data increases from VP1 to VP3 (see Appendix B). L-CUM = logistic model, cumulative probabilities; CM-CUM = coarsened multinomial model, cumulative probabilities; L-COND = logistic model, conditional probabilities; CM-COND = coarsened multinomial model, conditional probabilities. Results for estimators of treatment effect on perinatal transmission and intrapartum transmission are combined under the heading A2/A3.

#### Cumulative regression models

The proposed cumulative regression model (CM-CUM) performed comparably to, or better than, the logistic regression model (L-CUM) across all performance measures for all scenarios except where treatment was assumed to reduce the odds of in utero and intrapartum transmission by roughly equal amounts (TE1) and the visit process resulted in the least amount of missing data (VP1). For this scenario, the birth estimate was less biased in the logistic model.

The CM model performed most impressively for the TE4 scenarios. As the amount of missing test result data increased (from VP1 to VP3), the improvement offered by the CM model increased. Specifically, considering 4 to 8 weeks, the bias for the logistic model increased from 1.2-fold over the CM model under VP1 to 2.7- and 11.3-fold under VP2 and VP3, respectively.

Across all scenarios, MSE was consistently lower (albeit only slightly in most cases) for the CM model than for its logistic counterpart. The CPs for the competing cumulative models were similar while power at 4 to 8 weeks was higher for the CM model for all scenarios where power was assessed. The higher power observed at 4 to 8 weeks compared to birth is not surprising given the smaller probability of infection at birth.

#### Conditional regression models

On the whole, the proposed conditional regression model (CM-COND) performed comparably to its logistic counterpart (L-COND), with the CM model having less bias at birth for all scenarios where a positive effect of treatment on intrapartum and overall perinatal transmission was assumed (TE1, TE3, and TE4). MSE was consistently lower, although only slightly, for the CM model. While power tended to be low for the conditional models, power at birth and at 4 to 8 weeks was slightly higher for the CM model for the TE4 scenarios.

### HPTN 024

**Figure 1:**
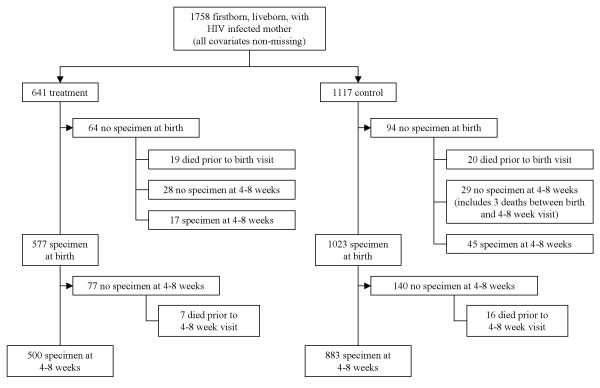
HPTN 024 testing profile.

Of 2,052 firstborn infants born alive to HIV positive mothers, 1,758 had complete data with respect to the covariates of interest. Of these, 1,696 had a test result at some point during follow-up and were included in the analysis of HIV infection. 1,739 had a test result or are known to have died during follow-up and were, thus, included in the analysis of HIV infection or death. Descriptive statistics for the 1,739 infants included in the analysis of HIV infection or death are provided in Table 3. Figure 1 provides the complete testing profile for the 1,758 infants with complete covariate data, according to treatment group.

**Table 3:** Descriptive statistics for 1,739 infants included in analysis of HIV infection or death.

Covariate	Treatment		Control	
		
	Mean/N	SD/%	Mean/N	SD/%
Maternal viral load (1 log10 unit)	4.338	0.836	4.242	0.817
CD4 count (100 units)	3.697	2.067	3.796	2.234
Female infant	296	47%	566	51%
Mother nevirapine	603	95%	1066	96%
Mother and infant nevirapine	558	88%	1001	90%

We used the proposed regression methods to analyze the outcomes infection and infection or death. We estimated the odds of perinatal transmission using the proposed cumulative model and the odds of in utero and intrapartum transmission using the proposed conditional model. Results are provided in Table 4. We found that treatment was not significantly associated with a reduction in any of the modes of PMTCT. These findings are consistent with the intent-to-treat analysis as is the trend in the estimates suggesting that treatment decreases the odds of in utero transmission while increasing the odds of perinatal transmission [11]. When we defined first positive test or death at or before a given visit as the endpoint, we observed the same trend, although slightly weaker.

**Table 4:** Adjusted odds ratios at birth, 4 to 8 weeks.

Risk factor	In utero		Perinatal		Intrapartum	
			
	OR	95% CI	OR	95% CI	OR	95% CI
Infection endpoint (612 treatment, 1084 control)						

Treatment vs. control	0.691	0.469, 1.017	0.987	0.736, 1.323	1.404	0.928, 2.123
Maternal viral load (1 log10 unit)	2.552	1.895, 3.437	2.820	2.221, 3.579	2.873	2.023, 4.082
CD4 count (100 units)	1.037	0.939, 1.145	1.099	1.015, 1.190	1.172	1.033, 1.331
Female infant	0.938	0.658, 1.339	1.011	0.764, 1.338	1.101	0.733, 1.654
Mother nevirapine	0.549	0.248, 1.215				
Mother and infant nevirapine			0.628	0.387, 1.019	0.562	0.262, 1.206

Infection/death endpoint (632 treatment, 1107 control)						

Treatment vs. control	0.873	0.632, 1.205	1.025	0.789, 1.332	1.245	0.850, 1.825
Maternal viral load (1 log10 unit)	2.299	1.786, 2.959	2.568	2.086, 3.161	2.597	1.895, 3.558
CD4 count (100 units)	1.043	0.959, 1.135	1.098	1.024, 1.178	1.175	1.048, 1.318
Female infant	0.883	0.650, 1.201	1.066	0.829, 1.370	1.336	0.916, 1.949
Mother nevirapine	0.221	0.128, 0.381				
Mother and infant nevirapine			0.452	0.302, 0.678	0.546	0.277, 1.075

## Discussion

Many statistical techniques are available for estimating PMTCT of HIV while adjusting for covariates. Among the more commonly used are logistic regression models and Cox proportional hazards models. While these methods are relatively straightforward to implement, they do not easily accommodate missed or unscheduled visits while allowing for a time-varying treatment effect. Cox models can be modified to allow the effect of treatment to depend upon time but do not fully solve the problem of how to handle missed or unscheduled visits. Interval censored models, which use an infant's time to last negative test and time to first positive test to form an interval around his or her (unknown) time of infection, may better accommodate the missing data, but software is not generally available for regression with interval censored data unless we are willing to make parametric assumptions about the distribution of the event times.

Recently, Bang and Spiegelman [12] proposed a likelihood approach for a dichotomous outcome to estimate mother to child transmission when infection status is missing for some infants due to fetal loss. However, this approach does not address all three of our endpoints of interest or missing data due to incomplete follow-up. Balasubramanian and Lagakos [13] provide methods for estimating the continuous distribution of the timing of in utero and peripartum transmission that account for the imperfect sensitivity of the HIV assay. The authors developed the approach for settings in which there is no risk for infection following birth and, therefore, do not address the potential impact of breastfeeding.

We propose a coarsened multinomial approach for estimating PMTCT that accommodates missing test result data, regression on the three outcomes of interest, and time-varying treatment effects. Through simulation, we investigated the performance of the estimators obtained from the more commonly used logistic regression approaches and compared them to the proposed estimators, including imperfect sensitivity of the assay and contamination of the endpoint due to early breastfeeding transmission. We found that both the proposed cumulative model and the proposed conditional model performed well when compared to their logistic counterparts. Performance of the proposed cumulative model was particularly strong under scenarios where treatment was assumed to increase the risk of in utero transmission but decrease the risk of intrapartum and overall perinatal transmission and under scenarios designed to represent interim analyses. Power for the proposed models was consistently higher at 4 to 8 weeks, which is to be expected given that the logistic models used only data for infants whose endpoints were non-missing or could be imputed based on subsequent negative tests.

The coarsened multinomial regression approach is not without limitations. Both the proposed cumulative and conditional models impose non-linear constraints on the coefficients, which can complicate interpretation of the estimates if maximization of the likelihood occurs on the boundary of the parameter space. In the case of the conditional model, however, only a single constraint is imposed, which is no more than would be imposed for a general multinomial model [[14], p. 21]. In numerous simulations (beyond those presented here), we saw no evidence of bias due to maximization on the boundary. Our approach relies on the assumption that missingness is non-informative and, thus, may be more appropriate for some endpoints (infection/death) than for others (infection). While the model can be used in a breastfeeding population, it does not allow us to separate intrapartum transmission from early transmission due to breastfeeding.

While we have attempted to assess the impact of misclassification in our simulations, our approach uses probabilities of first testing positive to estimate transmission probabilities and, in doing so, does not formally account for misclassification due to the imperfect sensitivity of testing. A possible extension of this approach would involve introduction of a latent variable representing an infant's true infection status. One could link an infant's probability of infection at a given time point to his or her coarsened test result via his or her complete (unobserved) test result and, in doing so, incorporate information about the sensitivity of testing in the manner of Magder and Hughes [15]. Given the multivariate nature of the outcome and missingness in the test result, such an extension would introduce considerable complexity.

## Conclusion

Here we have studied the problem of estimating the effect of treatment on PMTCT of HIV when outcome data are incomplete. We describe methods that give consistent and asymptotically normal estimators using maximum likelihood theory. Through simulation, we have shown that the proposed models outperform standard logistic models in terms of bias, mean squared error, coverage probability, and power under a range of treatment effect and visit process scenarios designed to reflect a PMTCT setting. Given their strong performance, coarsened multinomial regression models are to be preferred to standard logistic models for estimation of perinatal mother to child transmission of HIV, particularly when assays are missing or occur off-schedule for some infants.

## Competing interests

The authors declare that they have no competing interests.

## Authors' contributions

ERB conceived of the study and provided overall guidance, in addition to reviewing analyses and participating in the drafting of the manuscript. ERB was also the lead statistician for the HPTN 024 study. CCG drafted the manuscript, developed code to implement the proposed models, and carried out the analyses. Both authors read and approved the final manuscript.

## Pre-publication history

The pre-publication history for this paper can be accessed here:



## Supplementary Material

Additional file 1 Appendix A: Simulation of time of detectable infection. Click here for file

Additional file 2 Appendix B: Simulation of visit process and determination of test results. Click here for file
