# Supplementary material for: A coarsened multinomial regression model for perinatal mother to child transmission of HIV
Source: BMC Med Res Methodol. 2008 Jul 15;8:46. doi: 10.1186/1471-2288-8-46 (PMC2515333; doi:10.1186/1471-2288-8-46)
Supplement: Additional file 1 — Appendix A: Simulation of time of detectable infection. [file 1471-2288-8-46-S1.pdf]

Appendix A: Simulation of time of detectable infection

In this section, we describe how we simulated time of detectable infection, which was used together with information on visit timing (discussed in Appendix B) to determine a simulated infant's sequence of test results. First, we simulated mode of transmission as either in utero, during delivery or neither. In doing so, we assumed that timing of infection is subject to the effects of a binary and a continuous predictor. The binary predictor (X_1) acts as a treatment with potentially different effects in utero and in the peripartum period and was drawn from a Bernoulli distribution with probability 0.5. We simulated the continuous predictor (X_2) from a normal distribution with mean 4.3 and standard deviation 0.8 to mimic the observed distribution of log 10 viral load in the HPTN 024 data.

We simulated mode of transmission under two frameworks, one based on a cumulative regression model and the other based on a conditional regression model. We calculated an infant's probabilities of in utero infection, $A1_i$, and perinatal infection, $A2_i$, according to $A1_i = g^{-1}(X_i'\beta_1)$ and $A2_i = g^{-1}(X_i'\beta_2)$ with $\beta_1 = (\beta_{10}, \beta_{11}, \beta_{12})'$, $\beta_2 = (\beta_{20}, \beta_{21}, \beta_{22})'$, and $g(\cdot)$ the logit link. We calculated an infant's probability of intrapartum infection, $A3_i$, according to $A3_i = g^{-1}(X_i'\beta_{2|1^-})$ with $\beta_{2|1^-} = (\beta_{2|1^-,0}, \beta_{2|1^-,1}, \beta_{2|1^-,2})'$ and $g(\cdot)$ the logit link. We determined an infant's probability of infection during delivery as $A2_i - A1_i$ under the cumulative framework and as $A3_i \times (1 - A1_i)$ under the conditional framework. To simulate mode of infection, we used a multinomial distribution with probability of in utero infection, probability of delivery infection, and probability of neither in utero or delivery infection.

We allowed for imperfect sensitivity of the test by generating the time of detectable infection. This reflects the fact that an intrapartum transmission is unlikely to be detected at the birth visit. We generated each infant's time of detectable infection as number of days since birth. For infants who became infected in utero, we assigned a time of detectable infection equal to zero days. For infants who became infected during delivery, we generated time of detectable infection according to a uniform (0,14) distribution. An upper limit of 14 days was chosen to accommodate the lag time inherent in detecting HIV infection [1]. We also allowed the simulations to reflect additional positive test results at the 4 to 8 week visit due to breastfeeding. For infants infected neither in utero nor during delivery, we generated time of detectable infection according to an exponential distribution, which added an average of 30 infections to the 0 to 8 week period for the cumulative model and an average of 32 infections to the 0 to 8 week period for the

Table A.1: Simulation of time of detectable infection for each treatment effect scenario

			Cumulative				Conditional	
			Treatment		Control		Treatment	Control
Treatment Effect	β_{11}	β_{21}=$\beta_{2\|1^-,1}$	A1	A2	A1	A2	A3	A3
TE 1	-0.55	-0.54	0.03	0.11	0.05	0.18	0.05	0.09
TE 2	-0.55	0.00	0.03	0.18	0.05	0.18	0.09	0.09
TE 3	-0.02	-0.38	0.05	0.13	0.05	0.18	0.06	0.09
TE 4	0.27	-0.27	0.07	0.14	0.05	0.18	0.07	0.09

conditional model.

We simulated time of detectable infection under four treatment effect scenarios, denoted TE1 through TE4. Under the cumulative framework, we took (β_{10}, β_{12}, β_{20}, β_{22}) = (-4, 0.25, -2.6, 0.25) for each scenario while under the conditional framework, we took (β_{10}, β_{12}, $\beta_{2|1^-,0}$, $\beta_{2|1^-,2}$) = (-4, 0.25, -3.4, 0.25) for each scenario. The effects of treatment on in utero, perinatal, and intrapartum transmission, represented by β_{11}, β_{21}, and $\beta_{2|1^-,1}$ respectively, were allowed to vary across scenarios as described in Table A.1. For all scenarios, we assumed that treatment had the same effect on perinatal transmission (cumulative model) as on intrapartum transmission (conditional model), that is, we took β_{21}=$\beta_{2|1^-,1}$. Table A.1 also provides the probabilities of in utero, perinatal, and intrapartum transmission for the treatment and control groups for each treatment effect scenario when the continuous predictor is taken to be equal to its average value.

References

1. Balasubramanian R, Lagakos SW: **Estimation of the timing of perinatal transmission of HIV**. *Biometrics* 2001, **57**:1048–1058.
