# Supplementary material for: A coarsened multinomial regression model for perinatal mother to child transmission of HIV
Source: BMC Med Res Methodol. 2008 Jul 15;8:46. doi: 10.1186/1471-2288-8-46 (PMC2515333; doi:10.1186/1471-2288-8-46)
Supplement: Additional file 2 — Appendix B: Simulation of visit process and determination of test results. [file 1471-2288-8-46-S2.pdf]

Table B.1: Probability tested during/following each visit window for each visit process

Visit Process	Birth	Between birth and 4-8 weeks	4-8 weeks	After 4-8 weeks
VP 1	0.85	0.05	0.75[1]	0.80
VP 2	0.85	0.05	0.50	0.25
VP 3	0.50	0.05	0.25	0.10

[1]0.85 used if time of detectable infection 0.

Appendix B: Simulation of visit process and determination of test results

In simulating each infant's visit process, we considered two visit windows, birth and 4 to 8 weeks, and the corresponding periods birth, between birth and 4 to 8 weeks, 4 to 8 weeks, and after 4 to 8 weeks. We simulated whether or not an infant was tested during each of these periods using a binomial distribution according to three visit process scenarios, denoted VP1 through VP3 (Table B.1). VP1 corresponds to an analysis that might be done at the end of a study when all of the data that are expected have been collected. For VP2, we assumed that a smaller percentage of infants are tested at the 4 to 8 week visit and after the 4 to 8 week visit. For VP3, we reduced these percentages further, as well as the percentage of infants tested at birth. VP2 and VP3 were designed to represent interim analyses.

We assigned time of visit as number of days since birth. Time of birth visit was generated according to a multinomial distribution with probability 0.4 for days 0 and 1 and probability 0.04 for days 2 through 6. For visits between birth and 4 to 8 weeks, time of visit was assigned according to a multinomial distribution with probability 0.05 for all days. For visits during the 4 to 8 week visit window, time of visit was assigned according to a multinomial distribution with probability 0.04 for all days. For visits after 4 to 8 weeks, time of visit was assigned according to a multinomial distribution, with days 275 through 325 weighted more heavily (probability = 0.008) than other days in the period (probability = 0.002). Each infant was allowed at most one visit in each of the windows described.

Finally, we compared each infant's simulated time of detectable infection to his or her simulated visit times to generate the infant's vector of observed results (Y). In assessing infection after 4 to 8 weeks, we considered only simulated times of detectable infection before day 500.
